# Bioactive oxylipins in type 2 diabetes mellitus patients with and without hypertriglyceridemia

**DOI:** 10.3389/fendo.2023.1195247

**Published:** 2023-08-17

**Authors:** Yanan Xiao, Anne Pietzner, Nadine Rohwer, Adelheid Jung, Michael Rothe, Karsten H. Weylandt, Ulf Elbelt

**Affiliations:** ^1^Division of Medicine, Department of Gastroenterology, Metabolism and Oncology, University Hospital Ruppin-Brandenburg, Brandenburg Medical School, Neuruppin, Germany; ^2^Medical Department, Division of Psychosomatic Medicine, Campus Benjamin Franklin, Charité-Universitätsmedizin Berlin, corporate member of Freie Universität Berlin and Humboldt-Universität zu Berlin, Berlin, Germany; ^3^Faculty of Health Sciences, Joint Faculty of the Brandenburg University of Technology, Brandenburg Medical School and University of Potsdam, Potsdam, Germany; ^4^Department of Molecular Toxicology, German Institute of Human Nutrition Potsdam-Rehbruecke, Nuthetal, Germany; ^5^Lipidomix, Berlin, Germany

**Keywords:** oxylipins, type 2 diabetes mellitus, polyunsaturated fatty acids, controlled attenuation parameter, hypertriglyceridemia

## Abstract

**Objective:**

Dyslipidemia, in particular elevated triglycerides (TGs) contribute to increased cardiovascular risk in type 2 diabetes mellitus (T2DM). In this pilot study we aimed to assess how increased TGs affect hepatic fat as well as polyunsaturated fatty acid (PUFA) metabolism and oxylipin formation in T2DM patients.

**Methods:**

40 patients with T2DM were characterized analyzing routine lipid blood parameters, as well as medical history and clinical characteristics. Patients were divided into a hypertriglyceridemia (HTG) group (TG ≥ 1.7mmol/l) and a normal TG group with TGs within the reference range (TG < 1.7mmol/l). Profiles of PUFAs and their oxylipins in plasma were measured by gas chromatography and liquid chromatography/tandem mass spectrometry. Transient elastography (TE) was used to assess hepatic fat content measured as controlled attenuation parameter (CAP) (in dB/m) and the degree of liver fibrosis measured as stiffness (in kPa).

**Results:**

Mean value of hepatic fat content measured as CAP as well as body mass index (BMI) were significantly higher in patients with high TGs as compared to those with normal TGs, and correlation analysis showed higher concentrations of TGs with increasing CAP and BMI scores in patients with T2DM. There were profound differences in plasma oxylipin levels between these two groups. Cytochrome P450 (CYP) and lipoxygenase (LOX) metabolites were generally more abundant in the HTG group, especially those derived from arachidonic acid (AA), eicosapentaenoic acid (EPA), docosahexaenoic acid (DHA), γ-linolenic acid (γ-LA), and α-linolenic acid (α-LA), and a strong correlation between TG levels and plasma metabolites from different pathways was observed.

**Conclusions:**

In adult patients with T2DM, elevated TGs were associated with increased liver fat and BMI. Furthermore, these patients also had significantly higher plasma levels of CYP- and LOX- oxylipins, which could be a novel indicator of increased inflammatory pathway activity, as well as a novel target to dampen this activity.

## 1 Introduction

Incidence and prevalence of type 2 diabetes mellitus (T2DM) are rising worldwide with an alarming speed (1), leading to increased cardiovascular and metabolic morbidity and mortality. T2DM is a chronic condition characterized by hyperglycemia due to inadequate insulin secretion and/or defective insulin action. In 2001, McGarry proposed a new concept of glycolipid metabolism in diabetes mellitus, suggesting that excessive deposition of circulating free fatty acids (FFAs) and triglycerides (TGs) are key factors for the regulation of insulin action (2, 3). In this context, TG levels are an important biomarker of dyslipidemia, with adverse outcomes in patients with increasing hypertriglyceridemia (HTG) levels above 2 mmol/l (4).

Elevated TGs are common in T2DM and contribute to a two-fold increase in atherosclerotic cardiovascular disease (ASCVD) in T2DM. However, recommendations for dyslipidemia control in T2DM focus mostly on lowering low-density lipoprotein cholesterol (LDL-C) (5). The role of TG might be underappreciated though, as a recent study demonstrated that even an increase in TGs within the normal range confers an increased risk of T2DM in healthy subjects without metabolic syndrome (6). Furthermore, recent data confirm that elevated fasting TG (above 2.25 mmol/l) were associated with an increased risk of cardiovascular disease (CVD) mortality in patients with T2DM (7). Patients with T2DM and HTG also seem to have a more atherogenic lipoprotein phenotype than patients with elevated TG without T2DM (8)

The involvement of omega-3 (n-3) and omega-6 (n-6) polyunsaturated fatty acids (PUFAs) in the cardiovascular system and diabetes has been a subject of considerable interest. (9, 10). It has been shown that n-3 PUFAs can mediate biological effects in human cardiovascular, neurological, inflammatory diseases and cancer through different mechanisms such as alteration of membrane composition and function, gene expression and signaling molecules (11, 12). Notably, n-3 PUFA is also well-known for lowering TG levels (13) and liver fat content (14). However, the results of several recent large studies have been inconsistent in terms of cardiovascular benefit. The ORIGIN and STRENGTH trials observed moderate TG reductions but no impact on cardiovascular outcomes by n-3 PUFA supplementation (15, 16). A cardiovascular benefit was described with albeit minimal changes of TG in the GISSI-Prevenzione and JELIS trials (17, 18), and a significant 18% TG-decrease in the REDUCE-IT trial (19).

N-3 and n-6 PUFA derived oxylipins also have potent biological activity, with their metabolism relying on three enzymatic pathways including cytochrome P450 (CYP), cyclooxygenase (COX) and lipoxygenase (LOX), as well as non-enzymatic autoxidation (20, 21). The role of these oxylipins is multifaceted. Epoxidation products from n-3 PUFA are involved in regulating cardiac function, as well as reduce TG levels by suppressing hepatic lipogenesis and increasing fatty acid oxidation. (22, 23). In addition, evidence suggests that specialized pro-resolving mediators (SPM), derived from n-3 PUFA via LOX action, could regulate inflammation resolution (4). This supports the concept of n-3 PUFA derived inflammation-dampening oxylipins (n-3 IDOs) (25–27). In contrast, n-6 PUFA metabolites may have opposite or neutral effects (28). In a study involving 123 Caucasian men, researchers found that plasma concentrations of 5-hydroxyeicosatetraeonic acid (5-HETE) and 11-HETE were significantly higher, in obese individuals (29). This finding was further supported by evidence from the obese zucker mouse model (30), which elevated levels of 5-HETE, 12-HETE, and leukotriene B4 (LTB4) were discovered in adipocytes. Moreover, several studies revealed that 12- and 20-HETE may promote vasoconstriction, endothelial dysfunction, and platelet aggregation effects, which contribute to a higher risk of CVD (31, 32).

A scientific statement from the American Heart Association emphasized the need to lower triglycerides in order to lower CVD risk, with an optimal goal of < 1.1 mmol/l for fasting TG and a screening threshold of < 2.25 mmol/l (33). The proposed interventions focus on lifestyle to optimize diet, reduce weight and increase aerobic exercise (33) plus aggressive LDL-lowering therapies and control of hypertension, optimal glycemic control in individuals with T2DM and antithrombotic therapies for secondary prevention of ASCVD. In addition, icosapent ethyl is promoted in eligible patients (34).

On this background, we set out to analyze patients with T2DM to identify lipidomic changes that might modify liver and/or cardiovascular risk in patients with T2DM depending on their TG levels.

## 2 Methods

### 2.1 Subjects

Adult patients with T2DM were recruited from the gastrointestinal and endocrine clinics of Brandenburg Medical School, University Hospital Ruppin-Brandenburg from August 2020 to August 2021. Based on the normal range of TG values, the cohort was divided into the hypertriglyceridemia (HTG) group with TG ≥ 1.7 mmol/l (n = 22) and the control group with TG < 1.7 mmol/l (n =18).

The baseline characteristics including age, gender, and body mass index (BMI) were assessed, and the lifestyle of enrolled patients was obtained by standardized self-assessment and validated questionnaires. In addition, medical history and current medication were documented in the hospital records. Finally, we excluded individuals who were under 18 years of age and/or diagnosed with type 1, gestational or other specific types of diabetes. All patients gave written informed consent. The inclusion criteria for diabetic nephropathy (DN) were as follows: 1) diabetes history: the presence of type 2 diabetes with a duration of at least several years; 2) urinary markers and kidney function (at least one of the following conditions: urine albumin-to-creatinine ratio ≥ 30 mg/g or urine albumin excretion rate ≥ 30 mg/24 h (≥ 20 μg/min) on at least 2 out of 3 tests within 3-6 months; estimated glomerular filtration rate < 60/ml/1.73 m² for more than 3 months; 3) exclusion of other causes of chronic kidney disease. The study was approved by the local ethic committee of the Medical School Brandenburg (Number: Z-02-20170508), and the study was conducted in accordance with the principles of the Declaration of Helsinki.

### 2.2 Laboratory assessment

Venous blood was withdrawn from patients in the fasting state. Biochemistry parameters such as total cholesterol (TC), high-density lipoprotein cholesterol (HDL-C), TG, LDL-C, and glycated hemoglobin (HbA1c) were measured in a standard clinical setting by the central laboratory of the hospital.

### 2.3 Transient elastography

All patients underwent assessment of controlled attenuation parameter (CAP) (in dB/m) and liver stiffness measurement (LSM) (in kPa) by transient elastography (TE) (FibroScan, EchoSens). During performance of TE, patients were placed in a supine position, with the right arm raised behind the head, and then an ultrasound probe was used for site selection. Measurements were done from uniform liver parenchyma under the centerline of the probe, keeping the probe perpendicular to the skin. The TE testing procedure was performed by a skilled physician and was defined as a median of at least 13 valid measurements.

### 2.4 Fatty acid analysis

Blood samples were collected in EDTA tubes, centrifuged at 3500 rpm for 10 min at 4°C and the plasma samples were stored at -80°C until FAs analysis. Plasma samples for fatty acids composition analysis were prepared for gas chromatography (GC) according to established protocols for methylation and extraction of FAs (7, 35). For sample preparation, 100 µl of plasma was mixed with 50 µl pentadecanoic acid (PDA, 1 mg/mL in ethanol, Merck Schuchardt OHG, Hohenbrunn, Germany) as internal standard, 500 µl boron trifluoride (BF3, Sigma-Aldrich Chemie GmbH, Germany) in 14% methanol (Merck KGaA, Germany) and 500 µl n-hexane (Merck KGaA, Germany) in glass vials which were tightly closed. After vortexing, all samples were incubated at 100°C for 60 min in a preheated block. Then the mixture was added to 750 µl of water and vortexed for 4 minutes. After centrifugation, 100 µl of supernatant were transferred into a microinsert placed in a GC vial.

FAs analysis was performed using a 7890B gas chromatograph (Agilent Technologies, US) equipped with an HP88 column (112/8867, 60 m x 0.25 mm x 0.2 µm, Agilent Technologies, US) with the following temperature gradient: 50°C to 150°C with 20°C/min, 150°C to 240°C with 6°C/min, and 240°C for 10 min (total run time 30 min). Nitrogen was used as carrier gas at a flow rate of 1 ml/min. 1 µl of each sample was injected by splitless injection (injector 280°C). FID detection was performed at 250°C with the following flows: hydrogen at 20 ml/min, air at 400 ml/min, and make up at 25 ml/min. Methylated FA were identified by comparison of retention times with those of the Supelco^®^ 37 FAME mix (CRM47885, Sigma Aldrich, US) and a mix of single FAME standards [DPA, C22:5 n-3, AdA, C22:4 n-6 (Cayman Chemicals, Ann Arbor, MI, United States)]. Analysis and integration of peaks were carried out with OpenLAB CDS ChemStation Edition (Agilent Technologies, Santa Clara, CA, USA). Finally, the peak area of each group of FAs was calculated and, considering the individual response factors, the areas of the internal standard (PDA) were used to calculate the absolute levels of FA (μg/ml).

### 2.5 Oxylipin analysis

To analyze total (free and esterified) LOX- and CYP-derived metabolites, the sample preparation was performed based on a further developed protocol (20, 21), as follows. Plasma samples (500 μl) were spiked with a mixture of antioxidants and 100 pg each of deuterated internal standards 14,15-dihydroxyeicosatetraenoic acid (DHET)-D11, 15- HETE-d8, 20-HETE-d6, 8,9-epoxyeicosatrienoic acid (EET)-d11, 9,10-dihydroxy-octadecenoic acid (DiHOME)-d4, d4-12(13)-EpOME, d4-13- hydroxyoctadecadienoic acid (HODE), d4-prostaglandin E2 (PGE2) and LTB4-D4 (Cayman Chemical, Ann Arbor, MI). Methanol and sodium hydroxide were then added and alkaline hydrolysis was carried out at 60°C for 30 min. Following centrifugation and pH adjustment, the obtained supernatants were added to Bond Elute Certify II columns (Agilent Technologies, Santa Clara, USA) for solid phase extraction. The eluates were evaporated on a heating block at 40°C under a stream of nitrogen and the residues were dissolved in 100 µl methanol/water.

LC/ESI-MS/MS analysis was performed using an Agilent 1290 HPLC system with binary pump, multisampler and column thermostat equipped with a Zorbax Eclipse plus C-18, 2.1 x 150 mm, 1.8 µm column using a gradient solvent system of aqueous acetic acid (0.05%) and acetonitrile/methanol 50:50. The flow rate was set at 0.3 mL/min, the injection volume was 20 µL. The HPLC was coupled with an Agilent 6495 Triplequad mass spectrometer (Agilent Technologies, Santa Clara, USA) with electrospray ionization source. Analysis was performed with Multiple Reaction Monitoring in negative mode. One patient was excluded from the FA and oxylipin analysis due to incomplete blood components.

### 2.6 Statistical analysis

The collected data were analyzed with GraphPad Prism 9 (Graph Software, La Jolla, CA, USA). Data were tested for normal distribution and presented as mean with standard errors of the mean (SEM), and percentages (%). For comparisons between the two groups, the t-test was used for normally distributed data, the Mann-Whitney U-test for skewed distributed data, and the Chi-square test for categorical variables. Pearson/Spearman correlation was used to investigate the association between two groups of variables. *p* < 0.05 was considered as statistically significant.

## 3 Results

### 3.1 Study participants and biochemical parameters

A total of forty patients were included in the present study. The baseline characteristics, T2DM related complications and concomitant medication of the research participants are outlined in **Table 1**. Of these individuals, 20 were male and 20 were female, the mean age was 60.6 ± 1.13 years, and the mean BMI was 33.0 ± 1.07 kg/m^2^. However, patients with high TGs had significantly higher BMI values (35.0 ± 1.65 kg/m^2^) than those with normal TGs (30.7 ± 1.12 kg/m^2^). With a prevalence of up to 20%, CAD and DN were the most prevalent complications. In addition, eight patients had liver cirrhosis and two patients were diagnosed with chronic pancreatitis. Metformin (58%), insulin (50%) and aspirin (ASS) (43%) were the most commonly used drugs in this cohort of patients with T2DM. There was no statistical difference in the percentages of complications between the two groups. However, the proportion of prescribed ASS was significantly higher in the HTG group (61%).

**Table 1:** Clinical characteristics of the study.

Variables	Total (n=40)	TG (<1.7)	TG (≥1.7)	p
		n=18	n=22	
N (Female/Male)	20/20	10/8	10/12	0.53
Age (years)	60.6 ± 1.13	59.8 ± 1.04	61.3 ± 1.24	0.69
Weight (kg)	95.5 ± 2.91	90.1 ± 3.83	100.9 ± 4.00	0.06
Height (cm)	170.9 ± 4.5	169.7 ± 5.4	170.9 ± 5.2	0.72
BMI (kg/m^2^)	33.0 ± 1.07	30.7 ± 1.12	35.0 ± 1.65	0.04
Diabetic Complications, N (%)				
CAD	8 (20)	5 (28)	3 (14)	0.13
Stroke	1 (3)	1 (6)	0 (0)	–
PAD	3 (8)	1 (6)	2 (9)	0.86
Diabetic nephropathy	8 (20)	2 (11)	6 (27)	0.17
Diabetic neuropathy	2 (5)	1 (6)	1 (5)	0.73
Diabetic retinopathy	3 (7)	1 (6)	2 (9)	0.49
Diabetic foot syndrome	1 (3)	0 (0)	1 (5)	–
**Hepatic and pancreatic, N (%)**				
Liver cirrhosis	8 (20)	4 (22)	4 (18)	0.95
Chronic pancreatitis	2 (5)	1(6)	1 (5)	-
Medications, N (%)				
Metformin	23 (58)	11 (61)	12 (55)	0.83
DPP-IV-Inhibitor	12 (30)	6 (33)	6 (27)	0.68
SGLT-2-Inhibitor	7 (18)	4 (22)	3 (14)	0.77
Sulfonylurea	1(3)	1(6)	0 (0)	–
Acarbse	0 (0)	0 (0)	0 (0)	–
Glitazone	0 (0)	0 (0)	0 (0)	–
GLP-1-Analoga	4 (10)	1 (6)	3 (14)	0.75
Insulin	20 (50)	8 (44)	12 (55)	0.53
Statin	11 (28)	6 (33)	5 (23)	0.70
Fibrate	0 (0)	0 (0)	0 (0)	–
Ezetimib	3 (8)	2 (11)	1 (5)	0.86
Evolocumab	1 (3)	0 (0)	1 (5)	–
ACE-Hemmer	6 (15)	4 (22)	2 (9)	0.48
AT1-Receptor-Antagonist	16 (40)	7 (39)	9 (41)	0.90
Ca-Channel-Blocker	7 (18)	3 (17)	4 (18.2)	0.77
Beta-Blocker	13 (33)	4 (22)	9 (41)	0.21
Diuretics	11 (28)	5 (28)	5 (23)	0.75
ASS	17 (43)	11 (61)	6 (27)	0.03

Data are presented as mean ± SEM and number (percentage); p values are based on t-test or Chi-square test, and p < 0.05 was considered statistically significant; TG, triglyceride; BMI, body mass index; CAD, coronary artery disease; PAD, peripheral artery disease; ASS, aspirin; ‘-’: missing data.

**Figure 1:**
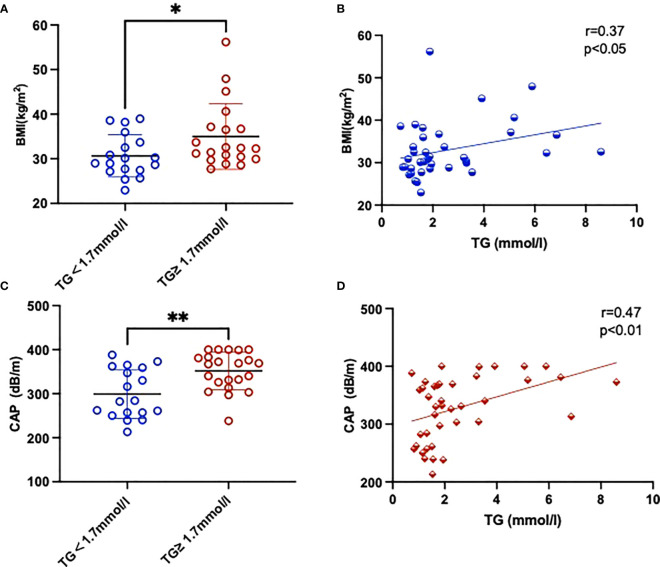
Comparison of BMI **(A)** and CAP **(C)** parameters between two groups with and without high TGs. Also shown is the correlation between BMI **(B)**, CAP **(D)** and TGs of the study. Results are shown as mean ± SEM. Statistics: **(A, C)** unpaired t-test/Mann-Whitney test, **(B, D)** Pearson/Spearman correlation. Significant changes are indicated as: *p < 0.05; **p < 0.01.

The clinical and laboratory features of patients are summarized in **Table 2**. We found that TC and LDL-C concentrations were higher in the HTG group than in the normal TG group whereas the levels of HDL-C were slightly lower, but these differences were not of statistical significance. The mean TG level in the HTG group was 3.54 ± 0.42 mmol/l, which was significantly higher than in the control group with 1.37 ± 0.07 mmol (*P* > 0.05). The mean CAP value in the HTG group was 345.7 ± 9.20 dB/m and thus significantly higher than in the normal TG group (299.2 ± 13.11 dB/m), whereas LSM values as indicator of fibrosis did not differ significantly. Furthermore, TG levels were positively correlated with BMI (r = 0.37, *P* < 0.05) as well as with CAP values (r = 0.47, *P* < 0.05), as shown in **Figure 1**.

**Table 2:** Laboratory and ultrasound characteristics of the study population.

Parameters	Total (n=40)	TG(<1.7)	TG(≥1.7)	p
		n=18	n=22	
HbA1c (%)	7.72 ± 0.27	7.88 ± 0.45	7.59 ± 0.37	0.67
TC (mmol/l)	5.00 ± 0.36	4.59 ± 0.29	5.35 ± 0.60	0.32
TG (mmol/l)	2.52 ± 0.29	1.37 ± 0.07	3.54 ± 0.42	<0.01
HDL-C (mmol/l)	1.13 ± 0.04	1.15± 0.06	1.11 ± 0.06	0.70
LDL-C (mmol/l)	3.08 ± 0.14	2.95 ± 0.22	3.19 ± 0.17	0.24
CAP (dB/m)	328.1 ± 8.74	299.2 ± 13.11	345.7 ± 9.20	<0.01
LSM (kPa)	10.44 ± 1.26	10.42 ± 2.21	10.45 ± 1.47	0.82

Data are presented as mean ± SEM; p values are based on t-test or Mann-Whitney test, and p < 0.05 was considered statistically significant; HbA1c, glycated hemoglobin; TC, total cholesterol; HDL-C, high-density lipoprotein cholesterol; LDL-C, low-density lipoprotein cholesterol; CAP, controlled attenuation parameter; LSM, liver stiffness measurement.

### 3.2 Plasma fatty acid levels

**Figure 2:**
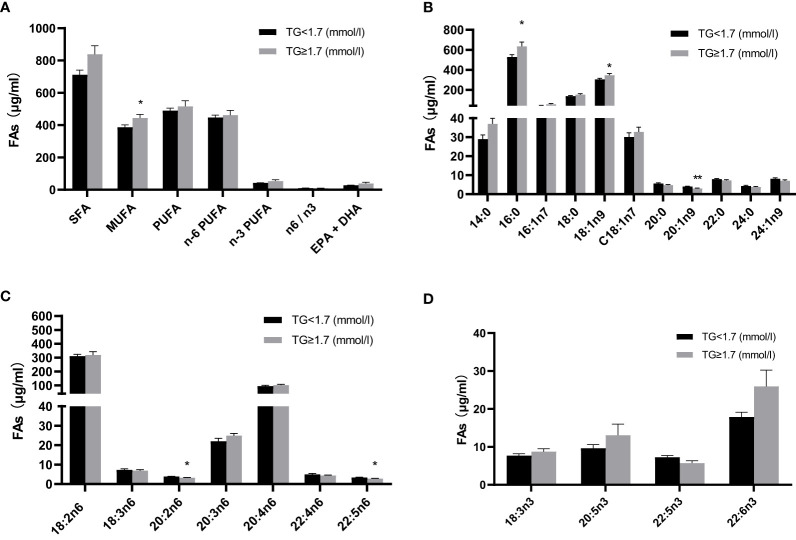
Comparison of absolute fatty acid levels (μg/ml) in plasma between two groups with and without high TGs. **(A)** Total fatty acids **(B)** individual fatty acids, with **(C)** n-6 fatty acids and **(D)** n-3 fatty acids. Results are shown as mean ± SEM. Statistics: unpaired t-test/Mann-Whitney test. Significant changes are indicated as: *p < 0.05; **p < 0.01. TG, triglyceride; SFA, saturated fatty acid; MUFA, monounsaturated fatty acid; PUFA, polyunsaturated fatty acid; EPA, eicosapentaenoic acid; DHA, docosahexaenoic acid.

Given that particularly n-3 PUFA have been shown to be able to affect TG levels we measured twenty-two FAs in plasma samples from the cohort assessed here (**Table 3**). FA content in plasma was dominated by saturated fatty acids (SFAs), followed by PUFAs and monounsaturated fatty acids (MUFAs) and the n-6 PUFAs constituted the largest fraction of total PUFAs. Compared with normal TG subjects, levels of C20:1n-9, C20:2n-6, and C22:5n-6 were significantly lower in HTG diabetics, whereas levels of C16:0, C18:1n-9c and MUFA were significantly higher in the HTG group. (**Figure 2**, **Table 3**).

**Table 3:** Absolute plasma fatty acids of the study.

Fatty Acid (μg/ml)	Total	TG (<1.7)	TG (≥1.7)	p
	n=40	n=18	n=22	
14:0	33.29 ± 2.56	28.93 ± 2.21	37.02 ± 2.85	0.21
16:0	585.78 ± 33.03	528.7 ± 22.14	634.7 ± 42.36	0.04
16:1n-7	49.12 ± 5.18	41.01 ± 4.16	56.07 ± 6.05	0.05
18:0	144.22 ± 7.89	136.3 ± 5.74	151 ± 9.73	0.22
18:1n-9	326.61 ± 13.61	304.1 ± 10.43	345.9 ± 16.33	0.04
C18:1n-7	31.54 ± 2.28	30.11 ± 2.11	32.76 ± 2.43	0.42
18:2n-6 (LA)	315.04 ± 19.48	311 ± 12.86	318.5 ± 25.16	0.80
20:0	5.14 ± 0.32	5.62 ± 0.36	4.73 ± 0.29	0.06
18:3n-6	7.09 ± 0.5	7.33 ± 0.5	6.89 ± 0.5	0.54
18:3n-3	8.26 ± 0.64	7.69 ± 0.49	8.75 ± 0.77	0.27
20:1n-9	3.48 ± 0.17	4.02 ± 0.2	3.03 ± 0.15	< 0.01
20:2n-6	3.53 ± 0.17	3.85 ± 0.18	3.25 ± 0.16	0.02
22:0	7.52 ± 0.38	7.86 ± 0.33	7.22 ± 0.41	0.24
20:3n-6	23.55 ± 1.34	21.99 ± 1.53	24.89 ± 1.19	0.14
20:4n-6 (AA)	98.75 ± 5.95	95.3 ± 5.99	101.7 ± 5.91	0.45
20:5n-3 (EPA)	11.5 ± 2	9.67 ± 0.91	13.08 ± 2.95	0.31
24:0	4.02 ± 0.29	4.27 ± 0.35	3.8 ± 0.24	0.35
24:1n-9	7.52 ± 0.49	8.08 ± 0.53	7.04 ± 0.46	0.15
22:4n-6	4.69 ± 0.32	5.02 ± 0.44	4.41 ± 0.22	0.21
C22:5n-6	3.07 ± 0.15	3.37 ± 0.17	2.81 ± 0.14	0.01
22:5n-3 (DPA)	6.45 ± 0.54	7.29 ± 0.44	5.72 ± 0.62	0.05
22:6n-3 (DHA)	22.22 ± 2.9	17.89 ± 1.27	25.93 ± 4.31	0.10
SFA	779.98 ± 41.43	711.7 ± 28.49	838.5 ± 52.52	0.53
MUFA	418.26 ± 18.44	387.3 ± 14.46	444.8 ± 21.86	0.04
PUFA	504.18 ± 25.8	490.4 ± 14.82	516 ± 35.22	0.53
n-6 PUFA	455.76 ± 22.05	447.9 ± 14.01	462.5 ± 28.94	0.67
n-3 PUFA	48.43 ± 5.14	42.53 ± 1.45	53.49 ± 8.3	0.24
n-6/n-3	10.23 ± 0.49	10.63 ± 0.34	9.89 ± 0.62	0.32
EPA + DHA	33.72 ± 4.52	27.55 ± 1.4	39.01 ± 7.19	0.11

Data are presented as mean ± SEM; p values are based on t-test or Mann-Whitney test, and p < 0.05 was considered statistically significant; LA, linoleic acid; AA, arachidonic acid; EPA, eicosapentaenoic acid; DPA, docosapentaenoic acid; DHA, docosahexaenoic acid; SFA, saturated fatty acids; MUFA, monounsaturated fatty acid; PUFA, polyunsaturated fatty acid.

### 3.3 Oxylipin levels

**Figure 3:**
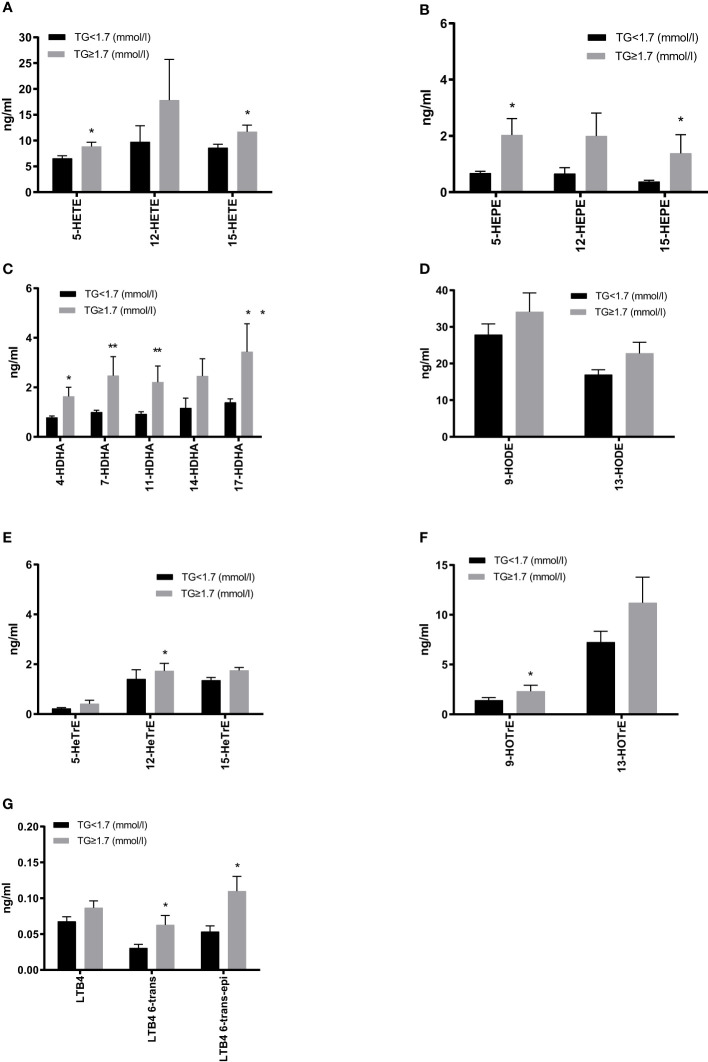
Comparison of plasma 5-lipoxygenase (LOX), 12-LOX and 15-LOX metabolites derived from arachidonic acid (AA) **(A)**, EPA **(B)**, DHA **(C)**, γ-linolenic acid (γ-LA) **(D)** and the LOX metabolites level derived from LA **(E)** and α-LA **(F)**, as well as leukotrienes derived from AA **(G)**. All results are shown as the mean ± SEM, p values are based on t-test or Mann-Whitney test; Significant changes are indicated as: *p < 0.05; **p < 0.01; HETE, hydroxyeicosatetraenoic acid; HEPE, hydroxyeicosapentaenoic acid; HDHA, hydroxydocosahexaenoic acid; HODE, hydroxyoctadecadienoic acid; HETrE, hydroxyeicosatrienoic acid; HOTrE, hydroxyoctadecatrienoic acid; LTB4, leukotriene B4.

We next assessed the plasma oxylipin profiles between T2DM subjects with and without HTG. The linoleic acid (LA) metabolites were the most abundant, followed by those of arachidonic acid (AA). Compared to the normal TG group, products of the LOX pathway were generally higher in patients with HTG. Our results show that 5-/15-HETE, 5-/15-hydroxyeicosapentaenoic acid (HEPE), and 4-/7-/11-/17-hydroxyeicosapentaenoic acid (HDHA) are substantially increased in HTG patients, as well as the 8-/10-/13-/16-HDHA metabolites. Moreover, LA, γ-linolenic acid (γ-LA) and α-linolenic acid (α-LA) produced oxylipins HODE, hydroxyeicosatrienoic acid (HeTrE) and hydroxyoctadecatrienoic acid (HOTrE), were all higher, and the 12-HeTrE and 9-HOTrE reaching significance, in the HTG group (**Figures 3A–F**, **Supplementary Table 1**). AA 5-LOX -derived LTB4 also showed an increasing trend, and the non-enzymatic hydrolysis LTB4-6-trans and LTB4-6-trans-epi levels were significantly higher in the HTG group than in the normal TG group. (**Figure 3G**, **Supplementary Table 1**).

**Figure 4:**
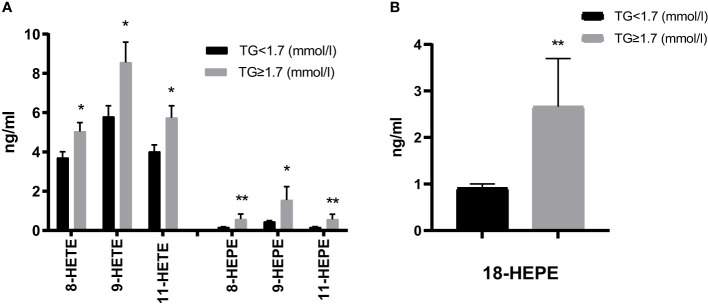
Comparison of non-enzymatic oxidation (AA, EPA) products **(A)** and the EPA-derived E-resolvin precursor 18-HEPE **(B)**. Results are shown as mean ± SEM. Statistics: unpaired t-test/Mann-Whitney test. Significant changes are indicated as: *p < 0.05; **p < 0.01.

Furthermore, non-enzymatic monohydroxy products were increased in the HTG group, with significant differences for AA and EPA-derived 8-/9-/11-HETE and 8-/9-/11-HEPE. The largest increases were observed in 9-HETE. Additionally, 18-HEPE (described as resolvin E precursor) was significantly more abundant in the HTG group (**Figure 4**, **Supplementary Table 1**).

**Figure 5:**
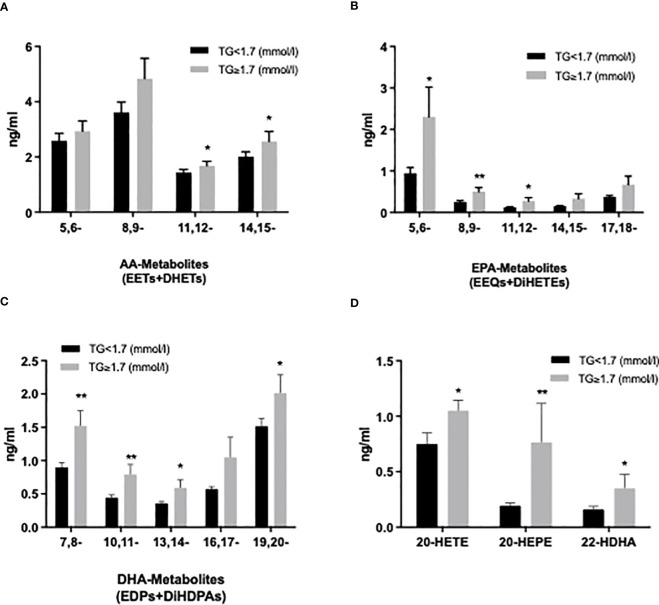
Comparison of plasma cytochrome P450 (CYP) epoxygenase metabolites level derived from AA **(A)**, EPA **(B)**, DHA **(C)**, as well as CYP450 ω-hydroxylase metabolites level of AA (20-HETE), EPA (20-HEPE), and DHA (22-HDHA) **(D)**. All results are shown as the mean ± SEM, P values are based on t-test or Mann-Whitney test; Significant changes are indicated as: *p < 0.05; **p < 0.01; EET: epoxyeicosatrienoic acid; DHET: dihydroxy-eicosatetraenoic acid; EEQ: epoxyeicosatetraenoic acid; DiHETE: dihydroxy-eicosatetraenoic acid; EDP: epoxydocosapentaenoic acid; DiHDAP: dihydroxy-docosapentaenoic acid; EpOME: epoxyoctadecamonoenoic acid; DiHOME: dihydroxy-octadecenoic acid.

We also evaluated the CYP and sEH pathway products in plasma from both groups focusing here on AA-, LA-, EPA-, DHA-derived epoxy-metabolites, the epoxyeicosatrienoic acids (EETs), epoxyoctadecamonoenoic acids (EpOMEs), epoxyeicosatetraenoic acids (EEQs) and epoxydocosapentaenoic acids (EDPs), which can be further converted to DHETs, DiHOMEs, dihydroxy-octadecenoic acids (DiHETEs) and dihydroxy-docosapentaenoic acids (DiHDPAs) by soluble epoxide hydrolase (sEH). The levels of 5,6-/8,9-EEQ, 8,9-/11,12-DiHETE, 7,8-/10,11-/13,14-/16,17-/19,20-EDP and 10,11-/13,14-/16,17-DiHDPA were significantly higher in HTG group. Similar changes were seen by summarizing the CYP-derived epoxy and sEH-derived dihydroxy products as shown in **Figures 5A–C**. Among the ω-hydroxylase metabolites, 20-HETE, 20- HEPE, 20-/22-HDHA derived from AA, EPA, and DHA were significantly increased in the HTG group. However, there were no significant differences in LA-CYP450 products between the two groups (**Figure 5D**, **Supplementary Table 1**).

**Figure 6:**
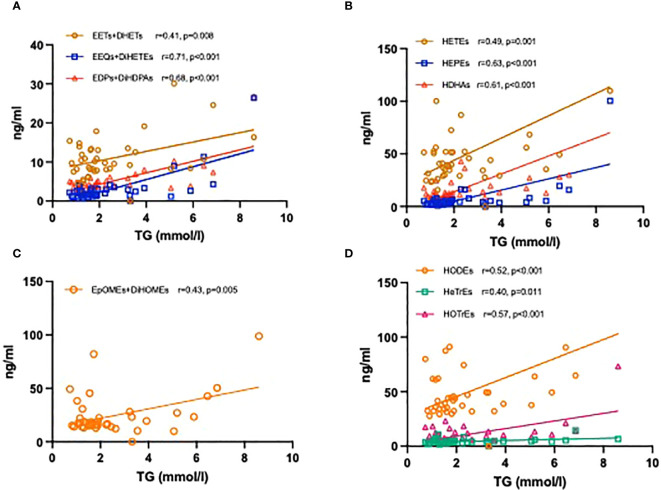
Correlation coefficients between the levels of TG and plasma oxylipins. CYP450 epoxygenase metabolites derived from AA, EPA, and DHA with TG levels **(A)**; LOX- and CYP450 ω-hydroxylase metabolites derived from AA, EPA, and DHA with TG levels **(B)**; CYP450 epoxygenase metabolites derived from LA with TG levels **(C)**; LOX- metabolites derived from LA, GLA and ALA with TG levels **(D)**.

Lastly, we investigated the relationship between levels of plasma oxylipins and TGs. Significantly positive correlations were found for the CYP epoxygenase and their corresponding sEH products with an r of 0.71 for EEQs+DiHETEs. In addition, a total of LOX- and CYP450 ω-hydroxylase products derived from AA, EPA, DHA, LA, GLA and ALA were also positively correlated with TG levels (**Figure 6**).

## 4 Discussion

In this study, we found significant differences in BMI and CAP values for patients with T2DM and high TGs (TG ≥ 1.7mmol/l) as compared to those with TGs within the reference range (TG < 1.7mmol/l), but no increase in liver fibrosis parameters. Hypertriglyceridemia was strongly associated with an increased BMI, as well as increased CAP values in patients with T2DM. Furthermore, total amount of palmitic and oleic acid was significantly increased in blood plasma from diabetics with increased TGs. We also observed significant changes in plasma oxylipin profiles between these two groups. Metabolites tended to be higher in hypertriglyceridemic patients, possibly indicating a (subclinical) pro-inflammatory environment arising from the hypertriglyceridemic metabolic dysregulation.

Our findings regarding HTG and liver fat content are consistent with other findings. In a multivariate analysis of nearly 300 patients with and without NAFLD, TGs were strongly associated with liver fat content (36). In addition, this research also indicated that CAP values of patients with T2DM progressively increased with rising TG levels. A recent large trial by de Lédinghen et al. analyzed patients with suspected chronic liver disease (37) and demonstrated that CAP values significantly increased with an increasing pathology of metabolic syndrome (MetS) components.

Pathophysiological mechanisms of MetS are complex and manifold. FFAs and lipid toxicity lead to dysfunction of cell membrane structure, inhibition of glycogen synthesis, and an increase of IR (38). Moreover, fat accumulation promotes inflammation and a thrombotic state that exacerbates atherosclerosis (39). The relationship between elastography measurements and NAFLD has been extensively documented in clinical trials (40, 41). In summary, our data confirm that CAP levels are closely related to MetS components. In clinical practice, determination of MetS patients relies on clinical examination and blood tests. However, based on the non-invasive, rapid, simple, and reproducible characteristics of CAP, adding it to the clinical assessment of MetS development might improve detection and risk stratification. Even inclusion of NAFLD into the definition of the metabolic syndrome could be a step to better identify the population with metabolic risk (42).

As the n-3 PUFA have been proven to lower TGs and reduce cardiovascular risk (19), studies have shown that n-6 and n-3 PUFA serum levels are negatively correlated with TGs [e.g. (43)] and that n-3 PUFA administration lowers triglycerides also in healthy volunteers (20), as well as in patients (44). Both higher serum n-6 and n-3 PUFA were associated with a lower risk of NAFLD (45). However, our data do not show these inverse relationships between n-3 and n-6 PUFAs observed elsewhere. This might be due to the fact that we measured plasma and not serum samples. Indeed, except for significantly higher levels of palmitic acid, which is known to promote insulin resistance in T2DM patients (46), oleic acid, and lower levels of γ-LA, and 11-eicosenoic acid, we did not find significant differences, and not even a trend toward higher levels of n-3 and n-6 PUFAs in the high TG (and high CAP) group. Further investigation may be needed to consider not only different types of samples but also other potential factors contributing to the observed differences, such as variations in study populations, dietary habits, or methodology. This could help clarify the relationship between n-3 and n-6 PUFAs and TG levels.

As shown in the present study, monohydroxy metabolites of PUFAs were significantly higher in those patients with high TGs than in those with normal TGs, with a broad range of AA- as well as EPA- and DHA- metabolites displaying significant increases. In contrast, in the study by Schuchard et al. there were only slightly higher serum concentrations of 5-HEPE, a lower concentration of 12-HETE in hyperlipidemic patients compared to normolipidemic patients (47). In this population diabetes was not studied, though.

Increased TG levels can provide more FAs substrates for oxylipin production. Therefore, as TGs are broken down, they release FAs that can be converted into oxylipins through enzymatic oxidation (26). Recent research has further supported this observation, indicating a linear relationship between the generation of oxylipins and triglyceride oxidation (48). Meanwhile, gene expression also appears to influence the route of PUFA metabolism. Several studies have indicated that high TGs activate PPAR-alpha, leading to increased expression of genes involved in beta-oxidation and promoting the expression of inflammatory oxylipins (49). Moreover, excess TGs and their FFAs can cause accumulation of lipids, and then trigger the activation of stress-responsive signaling pathways. The activation of these pathways can lead to a comprehensive increase in the metabolites of PUFAs (50).

Oxylipins are bioactive lipids, and changes in their levels reflect underlying inflammation or metabolic disturbances. There have been recent attempts to use oxylipins for stratification of MetS patients, showing that levels of a number of mono- and dihydroxy as well as epoxy metabolites were higher in MetS (51). In another study, it was shown that 5-/8-/and 12-HETE were increased in obese subjects with low-grade inflammation. However, weight reduction over an 8-week intervention led to a significant reduction of these oxylipins (52). There is also work ongoing to use oxylipin for liver disease risk evaluation [reviewed in (53)]. A comprehensive study was conducted to assess oxylipins as progression indicators from NAFLD to NASH and observed increases in various oxylipin classes with advancing disease stage (54). Another study has previously demonstrated in a lipidomic analysis of NAFLD/NASH that significantly increased levels of AA-products such as 5-HETE, 8-HETE and 15-HETE and 11-HETE indicate progression towards NASH (55). In a study of children with NAFLD, hepatic epoxyeicosanoids significantly increased with higher grades of steatosis (56). Therefore, our lipidomic analysis may also provide valuable insights into the metabolic risk stratification of T2DM.

Additionally, it is well-established that elevated levels of CYP- and LOX-metabolites, such as HETEs and LTs leads to inflammation, oxidative stress, endothelial dysfunction, and peripheral vascular resistance, and are generally associated with increased CVD risk. (57). 20-HETE has been reported to be associated with vascular inflammation and injury, angiogenesis, which are crucial factors in the development and progression of CVD (58). 9-HETE as a marker of oxidative stress was elevated in patients with coronary artery disease (57). In contrast, EETs help dilate arteries and reduce inflammation, but their sEH products may negate the protective effects (59). Given the experimental data implicating n-3 PUFA derived inflammation-dampening oxylipins in the alleviation of metabolic liver disease (25–27, 60), our data presented here support a concept of optimizing n-3 PUFA levels by identifying individuals with fatty acid imbalance and evidence of excess inflammation, rebalancing oxylipin profiles could significantly impact outcomes. This could alleviate metabolic disease-associated inflammation due to increased n-3 inflammation dampening oxylipin (n-3 IDO) formation with increased TGs, and liver fat, in patients with T2DM. Our study has several limitations. The majority of patients were enrolled during presentation to our gastroenterology and endocrinology unit, ranging from routine to presentations due to comorbidities (see **Table 1**). Although in multivariate linear regression, the positive effect indicators for two groups of HTG and non-HTG patients with TG levels showed no significant between-group associations (**Supplementary Table 2**), there may still be some confounding factors in our results, which should be interpreted with caution. Specifically, first, our patients with a higher BMI and higher triglycerides showed slightly better blood glucose control, which may be contrary to the experience in clinical practice. Second, there was probably some undertreatment with regard to guideline-based therapies, as evidenced by low prescription rates of statins. Last, our focus on diabetic patients in this study was based on the well-established association between diabetes and HTG, as well as a higher risk of cardiovascular disease. Future studies with more resources could expand the scope to investigate non-diabetic individuals as well.

## 5 Conclusion

The findings presented here indicate a clear association between TGs, BMI, and hepatic steatosis as measured by CAP in patients with T2DM. Compared to normal TG subjects, the unique feature of high TG subjects was the upregulation of CYP450 and LOX activity, accompanied by elevated oxylipin levels, potentially contributing to an inflammatory metabolic state.

## Data availability statement

The original contributions presented in the study are included in the article/**Supplementary Material**. Further inquiries can be directed to the corresponding authors.

## Ethics statement

The study involving human participants was reviewed and approved by Ethics Committee of Brandenburg Medical School. The patients/participants provided their written informed consent to participate in this study.

## Author contributions

Conceptualization: UE, YX, and KW. Methodology: AJ, MR, AP, NR, and KW. Analysis: YX, UE, and KW. Investigation: YX, UE, AJ, AP, and KW. Data curation: AJ, AP, and YX. Writing—original draft preparation: YX and KW. Writing—review and editing: YX, UE, and KW. Visualization: YX, and KW. Supervision: UE and KW. Project administration: UE and KW. All authors have read and agreed to the published version of the manuscript. All authors contributed to the article.
