# Supplementary material for: Bioactive oxylipins in type 2 diabetes mellitus patients with and without hypertriglyceridemia
Source: Front Endocrinol (Lausanne). 2023 Aug 17;14:1195247. doi: 10.3389/fendo.2023.1195247 (PMC10472135; doi:10.3389/fendo.2023.1195247)
Supplement: Supplementary file 1 [file Table_1.docx]

**Supplementary Materials**

| **Table S1.** Oxylipin composition of the study | | | | | |  |
| --- | --- | --- | --- | --- | --- | --- |
|  |  |  |  |  |  |  |
|  |  |  |  |  |  |  |
| **Fatty Acid** | **Enzyme** | ***Oxylipin*** | **Total** | TG（＜1.7） | TG（≥1.7） | **P value** |
|  |  |  | **n=40** | **n=18** | **n=22** |  |
| **AA** | **CYP (Epoxy-)** | **5,6-EET** | **0.64±0.07** | 0.57±0.06 | 0.72±0.07 | 0.125 |
|  |  | **8,9-EET** | 2.26±0.27 | 1.86±0.20 | 2.65±0.35 | 0.069 |
|  |  | **11,12-EET** | 0.84±0.09 | 0.75±0.07 | 0.94±0.11 | 0.183 |
|  |  | **14,15-EET** | 1.79±0.23 | 1.49±0.15 | 2.10±0.32 | 0.111 |
|  |  | **5,6-DHET** | 2.19±0.26 | 2.02±0.22 | 2.35±0.31 | 0.398 |
|  |  | **8,9-DHET** | 2.09±0.29 | 1.75±0.19 | 2.42±0.42 | 0.174 |
|  |  | **11,12-DHET** | 0.75±0.06 | 0.69±0.06 | 0.82±0.06 | 0.148 |
|  |  | **14,15-DHET** | 0.55±0.04 | 0.52±0.03 | 0.58±0.04 | 0.312 |
|  | **LOX** | **5-HETE** | 7.71±0.66 | 6.56±0.5 | 8.87±0.83 | **0.029** |
|  |  | **8-HETE** | 4.38±0.37 | 3.72±0.30 | 5.04±0.46 | **0.025** |
|  |  | **9-HETE** | 7.19±0.77 | 5.81±0.55 | 8.57±1.03 | **0.030** |
|  |  | **11-HETE** | 4.89±0.47 | 4.02±0.34 | 5.75±0.61 | **0.024** |
|  |  | **12-HETE** | 13.81±5.23 | 9.77±3.08 | 17.84±7.90 | 0.376 |
|  |  | **15-HETE** | 10.17±0.94 | 8.61±0.67 | 11.72±1.25 | **0.044** |
|  |  | **5-oxo-ETE** | 1.26±0.12 | 1.10±0.11 | 1.41±0.12 | 0.378 |
|  |  | **12-oxo-ETE** | 0.84±0.08 | 0.82±0.09 | 0.86±0.07 | 0.722 |
|  |  | **15-oxo-ETE** | 1.06±0.09 | 1.02±0.11 | 1.10±0.08 | 0.548 |
|  |  | **tetranor-12-HETE** | 0.12±0.02 | 0.08±0.01 | 0.16±0.03 | 0.262 |
|  | **CYP(Hydroxy-)** | **16-HETE** | 0.26±0.02 | 0.24±0.02 | 0.29±0.02 | 0.073 |
|  |  | **17-HETE** | 0.07±0.01 | 0.07±0.00 | 0.07±0.01 | 0.598 |
|  |  | **18-HETE** | 0.22±0.02 | 0.22±0.02 | 0.21±0.02 | 0.155 |
|  |  | **19-HETE** | 0.29±0.02 | 0.27±0.02 | 0.31±0.02 | 0.054 |
|  |  | **20-HETE** | 0.9±0.1.00 | 0.75±0.10 | 1.05±0.09 | **0.036** |
|  |  | **20-COOH-ARA** | 7.35±0.78 | 7.54±0.61 | 7.16±0.93 | 0.747 |
| **EPA** | **CYP (Epoxy-)** | **5,6-EEQ** | 0.01±0.01 | 0.03±0.01 | 0.00±0.00 | **0.011** |
|  |  | **8,9-EEQ** | 0.24±0.04 | 0.16±0.02 | 0.32±0.07 | **0.048** |
|  |  | **11,12-EEQ** | 0.16±0.03 | 0.09±0.01 | 0.22±0.07 | 0.106 |
|  |  | **14,15-EEQ** | 0.18±0.04 | 0.11±0.01 | 0.25±0.09 | 0.137 |
|  |  | **17,18-EEQ** | 0.29±0.08 | 0.15±0.01 | 0.44±0.18 | 0.153 |
|  |  | **5,6-DiHETE** | 1.67±0.38 | 0.92±0.14 | 2.41±0.74 | 0.074 |
|  |  | **8,9-DiHETE** | 0.15±0.02 | 0.10±0.01 | 0.20±0.04 | **0.019** |
|  |  | **11,12-DiHETE** | 0.05±0.01 | 0.04±0.00 | 0.07±0.02 | **0.017** |
|  |  | **14,15-DiHETE** | 0.06±0.01 | 0.05±0.00 | 0.08±0.02 | 0.093 |
|  |  | **17,18-DiHETE** | 0.25±0.03 | 0.23±0.02 | 0.27±0.04 | 0.364 |
|  | **LOX** | **5-HEPE** | 1.36±0.26 | 0.68±0.06 | 2.04±0.58 | **0.039** |
|  |  | **8-HEPE** | 0.37±0.10 | 0.17±0.02 | 0.58±0.26 | **0.006** |
|  |  | **9-HEPE** | 1.00±0.28 | 0.45±0.05 | 1.55±0.68 | **0.010** |
|  |  | **11-HEPE** | 0.37±0.10 | 0.17±0.02 | 0.57±0.25 | **0.007** |
|  |  | **12-HEPE** | 1.33±0.48 | 0.66±0.21 | 2.00±0.81 | 0.147 |
|  |  | **15-HEPE** | 0.88±0.26 | 0.38±0.04 | 1.38±0.66 | **0.012** |
|  | **CYP(Hydroxy-)** | **18-HEPE** | 1.79±0.43 | 0.92±0.09 | 2.67±1.03 | **0.005** |
|  |  | **20-HEPE** | 0.48±0.14 | 0.20±0.02 | 0.77±0.35 | **0.000** |
| **DHA** | **CYP (****Epoxy-)** | **7,8-EDP** | 0.70±0.09 | 0.46±0.04 | 0.93±0.15 | **0.008** |
|  |  | **10,11-EDP** | 0.36±0.06 | 0.24±0.02 | 0.49±0.11 | **0.039** |
|  |  | **13,14-EDP** | 0.34±0.05 | 0.24±0.02 | 0.45±0.10 | **0.009** |
|  |  | **16,17-EDP** | 0.60±0.09 | 0.42±0.03 | 0.77±0.17 | **0.006** |
|  |  | **19,20-EDP** | 0.76±0.14 | 0.50±0.04 | 1.02±0.29 | **0.006** |
|  |  | **7,8-DiHDPA** | 0.55±0.07 | 0.43±0.04 | 0.66±0.10 | 0.055 |
|  |  | **10,11-DiHDPA** | 0.28±0.04 | 0.21±0.03 | 0.34±0.05 | **0.010** |
|  |  | **13,14-DiHDPA** | 0.15±0.02 | 0.12±0.01 | 0.18±0.02 | **0.031** |
|  |  | **16,17-DiHDPA** | 0.21±0.04 | 0.15±0.01 | 0.26±0.08 | **0.042** |
|  |  | **19,20-DiHDPA** | 1.13±0.12 | 1.02±0.10 | 1.24±0.16 | 0.211 |
|  | **LOX** | **4-HDHA** | 1.21±0.18 | 0.78±0.06 | 1.64±0.37 | **0.040** |
|  |  | **7-HDHA** | 1.73±0.34 | 1.00±0.08 | 2.47±0.77 | **0.001** |
|  |  | **8-HDHA** | 1.84±0.34 | 1.06±0.09 | 2.63±0.77 | **0.001** |
|  |  | **10-HDHA** | 0.97±0.18 | 0.58±0.06 | 1.35±0.37 | **0.001** |
|  |  | **11-HDHA** | 1.57±0.31 | 0.92±0.10 | 2.21±0.65 | **0.001** |
|  |  | **13-HDHA** | 1.36±0.26 | 0.79±0.07 | 1.94±0.58 | **0.001** |
|  |  | **14-HDHA** | 1.81±0.57 | 1.17±0.40 | 2.46±0.70 | 0.237 |
|  |  | **16-HDHA** | 1.03±0.22 | 0.55±0.05 | 1.51±0.50 | **0.000** |
|  |  | **17-HDHA** | 2.42±0.52 | 1.39±0.14 | 3.44±1.13 | **0.003** |
|  | **CYP (Hydroxy-)** | **20-HDHA** | 4.02±0.74 | 2.54±0.23 | 5.51±1.51 | **0.001** |
|  |  | **22-HDHA** | 0.26±0.07 | 0.16±0.03 | 0.35±0.13 | **0.015** |
|  |  | **4-oxo-DHA** | 4.68±0.56 | 3.58±0.33 | 5.79±0.86 | **0.030** |
|  |  | **17-oxo-DHA** | 0.29±0.03 | 0.22±0.02 | 0.36±0.05 | **0.010** |
| **LA** | **LOX** | **9-HODE** | 31.00±3.97 | 27.88±2.92 | 34.12±5.16 | 0.329 |
|  |  | **13-HODE** | 19.87±2.12 | 16.97±1.34 | 22.77±3.06 | 0.073 |
|  |  | **13-oxo-ODE** | 1.05±0.11 | 0.96±0.08 | 1.15±0.15 | 0.313 |
|  |  | **9-oxo-ODE** | 5.95±0.6 | 5.17±0.26 | 6.74±1.02 | 0.109 |
|  | **CYP (Epoxy-)** | **9,10-EpOME** | 7.19±1.14 | 5.93±0.61 | 8.45±1.80 | 0.223 |
|  |  | **12,13-EpOME** | 10.15±2.18 | 8.24±1.52 | 12.06±2.97 | 0.204 |
|  |  | **9,10-DiHOME** | 3.56±0.32 | 3.98±0.32 | 3.15±0.30 | 0.276 |
|  |  | **12,13-DiHOME** | 3.77±0.40 | 3.83±0.50 | 3.71±0.30 | 0.834 |
| **γ-LA** | **LOX** | **5-HeTrE** | 0.32±0.08 | 0.23±0.03 | 0.42±0.14 | 0.349 |
|  |  | **8-HeTrE** | 1.04±0.08 | 0.91±0.09 | 1.16±0.06 | 0.111 |
|  |  | **12-HeTrE** | 1.58±0.34 | 1.41±0.37 | 1.74±0.30 | **0.013** |
|  |  | **15-HeTrE** | 1.56±0.11 | 1.36±0.11 | 1.76±0.12 | 0.266 |
| **α-LA** | **LOX** | **9-HOTrE** | 1.87±0.41 | 1.42±0.26 | 2.32±0.60 | **0.042** |
|  |  | **13-HOTrE** | 9.23±1.75 | 7.26±1.09 | 11.21±2.57 | 0.168 |
|  |  | **9-oxo-OtrE** | 0.25±0.03 | 0.18±0.01 | 0.31±0.04 | 0.135 |
| **AA** | **LOX** | **LTB4** | 0.08±0.01 | 0.07±0.01 | 0.09±0.01 | 0.135 |
|  |  | **LTB4 12-oxo** | 0.02±0.01 | 0.02±0.01 | 0.01±0.00 | 0.073 |
|  |  | **LTB4 6-trans** | 0.05±0.01 | 0.03±0.01 | 0.06±0.01 | **0.038** |
|  |  | **LTB4 6-trans-epi** | 0.08±0.01 | 0.05±0.01 | 0.11±0.02 | **0.021** |

Data are presented as mean ± SEM; p values are based on t-test or Mann-Whitney test, and p < 0.05 was considered statistically significant; EET: epoxyeicosatrienoic acid; DHET: dihydroxy-eicosatetraenoic acid; EEQ: epoxyeicosatetraenoic acid; DiHETE: dihydroxy-eicosatetraenoic acid; EDP: epoxydocosapentaenoic acid; DiHDAP: dihydroxy-docosapentaenoic acid; EpOME: epoxyoctadecamonoenoic acid; DiHOME: dihydroxy-octadecenoic acid; HETE: hydroxyeicosatetraenoic acid; HEPE: hydroxyeicosapentaenoic acid; HDHA: hydroxydocosahexaenoic acid;HETE: hydroxyeicosatetraenoic acid; HEPE: hydroxyeicosapentaenoic acid; HDHA: hydroxydocosahexaenoic acid; ETE: eicosatetraenoic acid; HODE:hydroxyoctadecadienoic acid; ODE:octadecadienoic acid; HETrE: hydroxyeicosatrienoic acid; HOTrE: hydroxyoctadecatrienoic acid; OTrE: octadecatrienoic acid; LTB4: leukotriene B4.

**Table S2.** Multiple Linear Regression Analysis Coefficients for TG Variables

|  | Unstandardized | | Standardized | *t* | p |
| --- | --- | --- | --- | --- | --- |
|  | *β* | SE | *β* |  |  |
| Variable |  |  |  |  |  |
| Gender | -0.39 | 0.61 | -0.10 | 0.63 | 0.54 |
| Age (years) | 0.01 | 0.03 | 0.05 | 0.30 | 0.77 |
| BMI | 0.07 | 0.06 | 0.24 | 1.10 | 0.28 |
| CAP | 0.01 | 0.01 | 0.30 | 1.46 | 0.15 |
| Liver cirrhosis | -0.96 | 0.88 | -0.20 | 1.09 | 0.28 |
| Chronic pancreatitis | -0.86 | 1.34 | -0.10 | 0.64 | 0.53 |
| ASS | -0.19 | 0.63 | -0.05 | 0.31 | 0.76 |

TG: triglyceride; BMI: body mass index; CAP: Controlled attenuation parameter; ASS: aspirin;
